# Biliary atresia in a preterm and extremely low birth weight infant: a case report and literature review

**DOI:** 10.1186/s40792-020-01092-5

**Published:** 2020-12-14

**Authors:** Yuki Kawano, Koichiro Yoshimaru, Yasuyuki Uchida, Keisuke Kajihara, Yukihiro Toriigahara, Takeshi Shirai, Yoshiaki Takahashi, Toshiharu Matsuura

**Affiliations:** Department of Pediatric Surgery, Graduate School of Medical Sciences, Kyushu University, 3-1-1 Maidashi, Higashi-ku, Fukuoka, 812-8582 Japan

**Keywords:** Biliary atresia, Preterm, Very low birth weight, Extremely low birth weight, Kasai portoenterostomy

## Abstract

**Background:**

Biliary atresia in very low birth weight (VLBW) and extremely low birth weight (ELBW) infants is rarely reported, and the optimal timing of Kasai portoenterostomy (KPE) in these cases remains unclear.

**Case presentation:**

We report a case of biliary atresia in a preterm female infant of 24 weeks of gestation who weighed 824 g. She underwent exploratory laparotomy and intraoperative cholangiography at 58 days of age (weight, 1336 g). Despite the diagnosis of biliary atresia with a type I cyst, we could only perform gallbladder drainage at that time due to the unstable intraoperative condition. While we waited for her body weight to increase, KPE was performed at 122 days of age (corrected age: 16 days), when the patient weighed 2296 g. Although she initially became jaundice-free, her liver function deteriorated due to cholangitis, and she developed decompensated cholestatic liver cirrhosis. Living donor liver transplantation was successfully performed at 117 days after KPE, and the postoperative course was uneventful. The timing of KPE is difficult to determine and a review of the relevant literature revealed that a poor prognosis in VLBW and ELBW infants with BA.

**Conclusions:**

Early KPE and careful postoperative follow-up, including liver transplantation is important for the improvement of outcomes.

## Background

Biliary atresia (BA) is a destructive inflammatory obliterative cholangiopathy of neonates that affects the intra- and extrahepatic bile ducts [1]. While the etiology remains unclear, BA is the most common cause of end-stage liver disease and is also the most frequent indication for liver transplantation (LT) in children [2, 3]. Preterm birth has been reported to be a poor prognostic factor in patients with BA [4, 5]. Although some studies have demonstrated the incidence and outcomes of BA in preterm infants, few studies have focused on BA in very low birth weight (VLBW) and extremely low birth weight (ELBW) infants. We herein report a case of BA in an ELBW preterm infant born at 24 weeks of gestation.

## Case presentation

A female preterm infant weighing 824 g was born to a 37-year-old woman at 24 6/7 weeks of gestational age (GA) by induced delivery due to chorioamnionitis. Because of bradycardia and unstable respiration, endotracheal intubation was performed immediately after birth, and surfactant was administered under the diagnosis of respiratory distress syndrome (RDS). The Apgar score was 3 at 1 min, 4 at 5 min. After admission to the NICU, the patient was put on mechanical ventilator support. At 4 days of age, a patent ductus arteriosus (PDA) was noted on echocardiography. Cardiac failure due to refractory PDA worsened in spite of the administration of catecholamine and diuretics. Therefore, PDA ligation was carried out at 24 days of age.

**Fig. 1:**
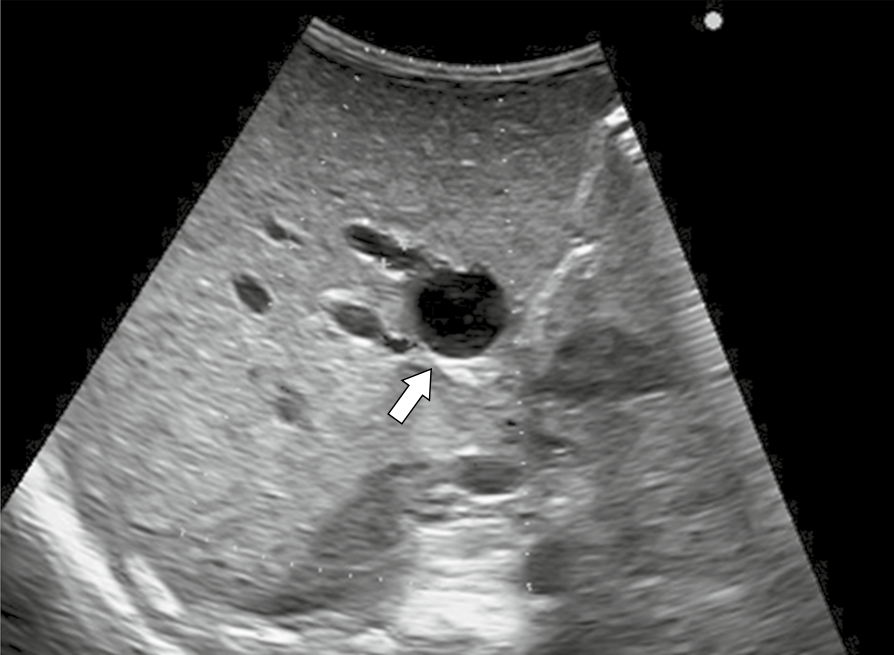
Ultrasonography at 37 days of age showed a biliary cyst at the porta hepatis (arrow)

**Fig. 2:**
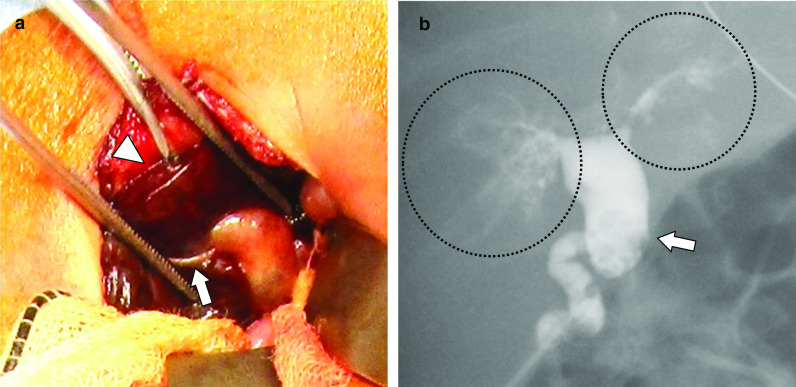
Intraoperative findings at 58 days of age (exploratory laparotomy and cholangiography). **a** The liver (arrowhead) was still soft. A drainage tube (arrow) was placed in the gallbladder. **b** Cholangiography revealed a cystic common bile duct (arrow) that had no communication with the duodenum and a cloudy pattern of intrahepatic ductules (circle)

At 15 days of age, mild hyperbilirubinemia with total bilirubin level of 3.9 mg/dL and a direct bilirubin level of 2.8 mg/dL was noted. Ursodeoxycholic acid (UDCA) and Inchinkoto (traditional Japanese herbal medicine) were administered; however, the conjugated hyperbilirubinemia worsened, and acholic stool was noted at 30 days of age. Abdominal ultrasonography showed a cystic structure at the porta hepatis with a diameter of 6.8 mm (Fig. 1); we therefore suspected BA with a type I cyst or congenital biliary dilatation (CBD). Since exploratory laparotomy (EL) and intraoperative cholangiography (IC) were essential for making a definitive diagnosis as early as possible, the operation was performed at 58 days of age, when the patient weighed 1336 g. During the operation, the liver was soft (Fig. 2a), and IC showed a cystic common bile duct that had no communication with the duodenum, with the intrahepatic ductules showing a cloudy pattern (Fig. 2b). These findings indicated BA with a type I cyst. Although we had initially planned to perform KPE for BA sequentially, we could only perform gallbladder drainage because the patient’s vital signs worsened during the operation. Since there were no significant bleeding or other intraoperative complications, the patient’s immaturity was considered to be the cause of this unstable condition.

**Fig. 3:**
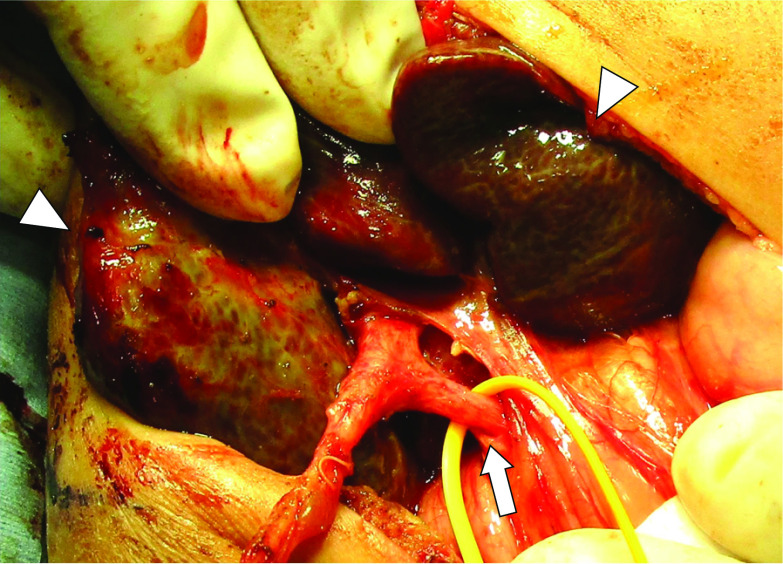
Intraoperative findings at 122 days of age (Kasai portoenterostomy). The liver (arrowhead) was firm and cirrhotic. The arrow indicates the common bile duct

**Fig. 4:**
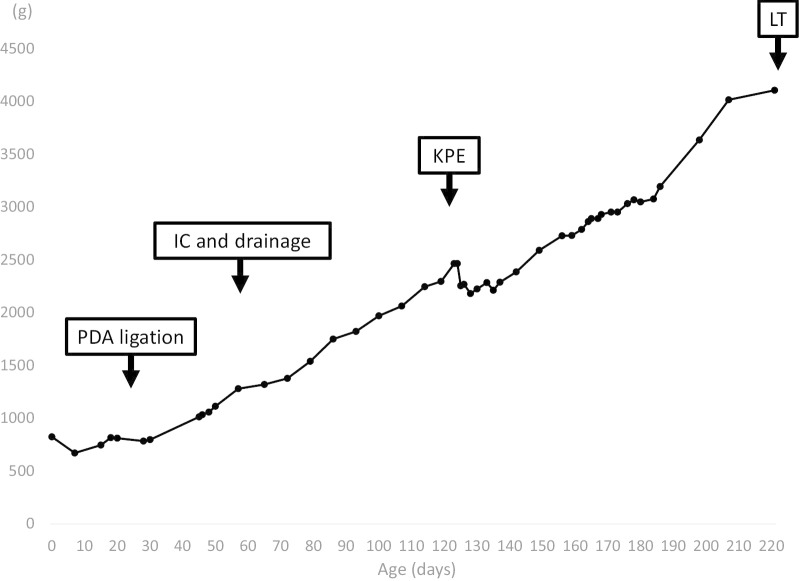
The trend in body weight. *PDA* patent ductus arteriosus, *IC* intraoperative cholangiography, *KPE* Kasai portoenterostomy, *LT* liver transplantation

After surgery, the color of the fluid drained from the gall bladder was yellowish, and the serum bilirubin level had improved but was not normal, which indicated that cyst-jejunostomy would be ineffective and that Kasai portoenterostomy (KPE) was necessary. We decided to wait for the patient to gain weight and to maintain her general condition. She was extubated at 72 days of age. At 122 days of age (corrected age: 16 days), we performed KPE, with the patient weighing 2296 g. The liver had become firm and cirrhotic (Fig. 3), and portoenterostomy with Roux-en-Y reconstruction was completed without complications. The examination of a liver biopsy revealed bridging fibrosis and cholestasis. The postoperative course was good, and prednisolone was administered from postoperative day (POD) 7 to 31 in accordance with our institution’s routine practice [6]. The serum bilirubin level normalized at POD 27. The patient’s body weight successfully increased (Fig. 4).

**Fig. 5:**
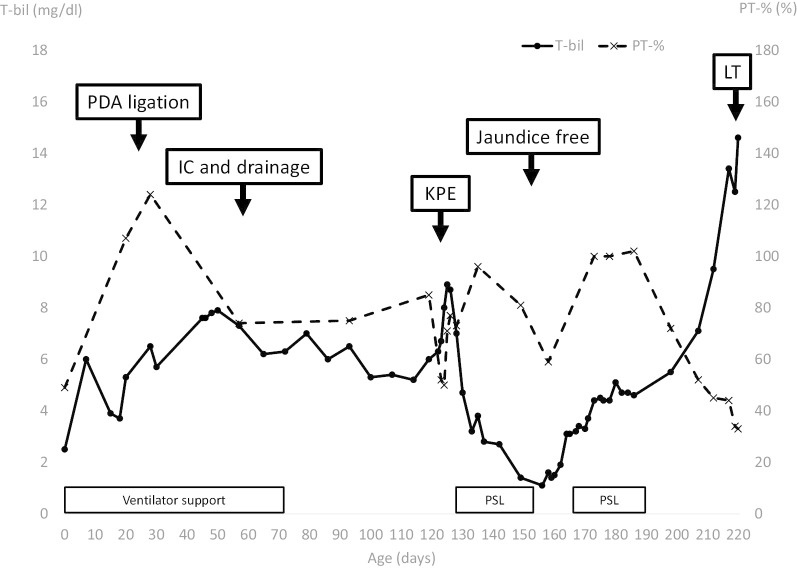
The trends in total bilirubin and PT-%. *PDA* patent ductus arteriosus, *IC* intraoperative cholangiography, *KPE* Kasai portoenterostomy, *LT* liver transplantation, *PSL* prednisolone

Although a jaundice-free status was initially achieved, the serum bilirubin, aspartate aminotransferase (AST), and alanine aminotransferase (ALT) levels began to increase following the development of cholangitis at 157 days of age (POD 35). Secondary prednisolone therapy was performed; however, the liver function continued to worsen (Fig. 5). Cholestasis, increasing ascites, coagulopathy, and gastrointestinal bleeding were noted subsequently with a pediatric end-stage liver disease (PELD) score 26 and Child–Pugh class C. At 221 days of age (corrected age: 133 days, 117 days after KPE), we performed living donor liver transplantation, with the patient weighing 4106 g. The donor was the patient's mother, who was 38 years of age and whose blood type was identical. The graft was a segment 2 (S2) monosegment graft and the graft-to-recipient weight ratio was 3.23%. Post-transplantation management was conducted as described in our previous report [7]. The postoperative course was uneventful, and the serum bilirubin and liver function profiles normalized by the first postoperative month. Currently, at 14 months after LT, the patient is well and shows a normal liver function with relatively good growth in height (-1.7 SD) and weight (-0.9 SD).

## Discussion

BA is a rare disease with an incidence of 1 in 8000–15,000 live births [1, 8]. According to the Japanese Biliary Atresia Registry (JBAR), 3,362 BA patients were identified in Japan from 1989 to 2017. Among them there were only 30 cases (0.89%) whose birth weight was less than 1500 g [9, 10]. Since these patients were rarely reported, the clinical course and therapeutic strategy remain unclear. We reported a case with a birth weight of 824 g, which is the lowest weight recorded for a BA patient in Japan.

In the present case, despite diagnosing the patient with BA by cholangiography at 58 days of age, we could not perform radical surgery at the time due to the unstable intraoperative condition. To determine the timing of KPE, we had to consider weight gain, the stabilization of the cardiac and respiratory condition, and the progress of treatment for PDA and RDS. We then performed KPE at 122 days of age (corrected age: 16 days). Eventually, the patient required LT due to the development of biliary cirrhosis at 3 months after KPE.

Preterm birth is reported to be a poor prognostic factor in BA; however, the exact mechanism remains unclear [4, 5, 11]. In general, the preoperative prognostic factors of KPE are the age at the operation, the presence of the biliary atresia splenic malformation (BASM) syndrome, liver histology, and the anatomic pattern of the bile ducts [12–14]. Most reports indicate that operating at an early age improves the prognosis [4, 12, 14]. Some studies have also investigated the ideal timing of KPE in preterm BA patients. Iwasaki et al. [15] recommended that surgery be performed by a corrected age of 60 days in preterm infants. Jiao et al. [4] also indicated, based on a retrospective study of 34 preterm BA patients, that KPE at a chronological age of ≤ 90 days and a corrected age of ≤ 60 days provided a good prognosis. On the other hand, a study of BA in preterm infants in Taiwan [11], which used the national registry system data, demonstrated that the mean corrected age at the time of KPE was 32.2 days (chronological age: 71.8 days) and that the native liver survival rate of preterm BA patients at 18 months of age was significantly lower than that of term infants (50% vs. 72.7%). This result suggests that the prognosis of preterm BA patients is poor, even if KPE is performed within a corrected age of 60 days.

Because few studies focused on BA in VLBW and ELBW infants, a review of the relevant literature was performed using PubMed and Ichushi-Web (a search engine for Japanese literature) to identify the characteristics and prognosis of these cases. Our search yielded 8 cases in the relevant English and Japanese literature; these cases and the present case are summarized in Table 1 [16–20]. All cases involved preterm birth, and the mean birth weight was 1123.8 g. Three cases were ELBW infants; our case had the lowest birth weight and was the only surviving ELBW infant among these reports. One case underwent primary LT, and the other seven underwent KPE. The mean body weight at KPE was 2385.2 g, probably because—in many of the cases—the operation was deferred empirically until a weight gain of 2000–2500 g was achieved. Although six cases underwent KPE within the corrected age of 60 days, five (83.3%) required subsequent LT in this review. The native liver survival rate of VLBW and ELBW infants with BA was found to be much poorer in comparison to general preterm BA patients, even though the KPE was performed within the corrected age of 60 days. Careful follow-up and avoiding delays in LT are necessary to improve the outcome.

**Table 1:** Cases of BA in VLBW and ELBW infants reported in the English and Japanese literature

Case	Authors	Year	Gestational age at birth (weeks)	Sex	Birth weight (g)	Chronological age at KPE (days)	Corrected age at KPE (days)^a^	Body weight at KPE (g)	Type of BA	Clinical course
1	Chen et al. [16]	2007	31	M	1375	51	(GA 38 2/7)	2164	III	LT at 7 months after KPE
2	Hashimoto et al. [17]	2007	27	F	834	187	96	2660	III-b1-ν	Died at 36 days after KPE
3	Fallon et al. [18]	2013	27	M	1090	107	20	N/A	N/A	LT at 2 years of age
4	Sanmoto et al. [19]	2017	33	F	1425	37	(GA 38 5/7)	2151	I-b1-β	Native liver survival at 5 months after KPE
5	Miyatake et al. [20]	2018	31	F	940	102	44	2346	N/A	LT at 9 months after KPE Died at 1 month after LT
6			29	F	1200	N/A	N/A	N/A	N/A	Primary LT at 7 months of age
7			33	F	1302	70	21	2694	N/A	LT at 8 months after KPE
8	Present case	2020	24	F	824	122	16	2296	I-cyst	LT at 3 months after KPE

*KPE* Kasai portoenterostomy, *BA* biliary atresia, *LT* liver transplantation, *GA* gestational age

^a^When the corrected age at KPE is a negative value, the gestational age (in weeks) is shown

The poor prognosis of KPE may imply the insufficiency of the criterion of the corrected age of 60 days. Given that recent studies have suggested that BA only manifests after loss of the protective physiology of the mother and placenta [2], the chronological age at KPE may reflect the prognosis more accurately rather than the corrected age. Actually, in our case, despite the liver being relatively soft at the first surgery (EL and IC) at 58 days of age, it became cirrhotic by the time of the second surgery (KPE) at 122 days of age. On the other hand, however, when KPE is performed too early, before sufficient weight gain can be achieved is associated with an increased risk of intraoperative and postoperative complications. Further studies with a larger sample size, which focus on BA in VLBW and ELBW infants should be performed to clarify the optimal timing of KPE in these cases.

## Conclusions

We described the clinical course and prognosis of BA in VLBW and ELBW infants. It is preferable to perform KPE earlier; however, the optimal timing is difficult to determine due to their complicated clinical condition. Because of the poor prognosis of KPE, careful postoperative follow-up, including LT, are important for improving patient outcomes.

## Data Availability

The datasets supporting the conclusions of this article are included within the article.

## Abbreviations

BA: Biliary atresia
LT: Liver transplantation
VLBW: Very low birth weight
ELBW: Extremely low birth weight
GA: Gestational age
RDS: Respiratory distress syndrome
PDA: Patent ductus arteriosus
UDCA: Ursodeoxycholic acid
CBD: Congenital biliary dilatation
EL: Exploratory laparotomy
IC: Intraoperative cholangiography
KPE: Kasai portoenterostomy
POD: Postoperative day
AST: Aspartate aminotransferase
ALT: Alanine aminotransferase
PT: Prothrombin time
PELD: Pediatric end-stage liver disease
JBAR: Japanese Biliary Atresia Registry
BASM: Biliary atresia splenic malformation
